# Pam2CSK4-adjuvanted SARS-CoV-2 RBD nanoparticle vaccine induces robust humoral and cellular immune responses

**DOI:** 10.3389/fimmu.2022.992062

**Published:** 2022-12-09

**Authors:** Yidan Qiao, Yikang Zhan, Yongli Zhang, Jieyi Deng, Achun Chen, Bingfeng Liu, Yiwen Zhang, Ting Pan, Wangjian Zhang, Hui Zhang, Xin He

**Affiliations:** ^1^ Institute of Human Virology, Department of Pathogen Biology and Biosecurity, Key Laboratory of Tropical Disease Control of Ministry of Education, Zhongshan School of Medicine, Sun Yat-sen University, Guangzhou, China; ^2^ Center for Infection and Immunity Study, School of Medicine, Sun Yat-sen University, Shenzhen, Guangdong, China; ^3^ Department of Medical Statistics, School of Public Health, Sun Yat-sen University, Guangzhou, Guangdong, China; ^4^ Guangzhou National Laboratory, Guangzhou, Guangdong, China

**Keywords:** SARS-CoV-2, nanoparticle vaccine, adjuvant, tuberculosis vaccine, TLR2 agonist, Pam2CSK4

## Abstract

As the global COVID-19 pandemic continues and new SARS-CoV-2 variants of concern emerge, vaccines remain an important tool for preventing the pandemic. The inactivated or subunit vaccines themselves generally exhibit low immunogenicity, which needs adjuvants to improve the immune response. We previously developed a receptor binding domain (RBD)-targeted and self-assembled nanoparticle to elicit a potent immune response in both mice and rhesus macaques. Herein, we further improved the RBD production in the eukaryote system by *in situ* Crispr/Cas9-engineered CHO cells. By comparing the immune effects of various Toll-like receptor-targeted adjuvants to enhance nanoparticle vaccine immunization, we found that Pam2CSK4, a TLR2/6 agonist, could mostly increase the titers of antigen-specific neutralizing antibodies and durability in humoral immunity. Remarkably, together with Pam2CSK4, the RBD-based nanoparticle vaccine induced a significant Th1-biased immune response and enhanced the differentiation of both memory T cells and follicular helper T cells. We further found that Pam2CSK4 upregulated migration genes and many genes involved in the activation and proliferation of leukocytes. Our data indicate that Pam2CSK4 targeting TLR2, which has been shown to be effective in tuberculosis vaccines, is the optimal adjuvant for the SARS-CoV-2 nanoparticle vaccine, paving the way for an immediate clinical trial.

## Introduction

Coronavirus disease 2019 (COVID-19), caused by SARS-CoV-2 virus infection, has been spreading around the world with more than 633 million positive infection cases and 6.6 million deaths (by the middle of November 2022, https://covid19.who.int/). The vaccine is considered an essential strategy for preventing the epidemic of infectious diseases (1). In the design of SARS-CoV-2 vaccines, full-length S1 subunit or Receptor Binding Domain (RBD) of Spike (S) protein is often chosen as the immunogen for most current vaccines. The S protein also plays a necessary role in mediating virus entry into host cells *via* interactions with angiotensin-converting enzyme-2 (ACE2) (2). Further studies have found that neutralizing monoclonal antibodies against Spike/RBD elicited by virus infection or vaccination can potently prevent the transmission of SARS-CoV-2 (3, 4). So far, more than thirty vaccines have been approved for clinical use worldwide (5).

Due to the low immunogenicity of inactivated or subunit vaccines, adjuvants are applied to benefit the immune response by improving antigen presentation, recognition, immune reaction type, immune cell activation, proliferation, and so on (6–9). In the early stage of adjuvant development, alum and emulsion were majorly used to enhance vaccine efficacy by controlled release, improving antigen-presentation, and modulating the adaptive immune system. Lately, adjuvants targeting the key innate immune signals, such as Toll-like receptor (TLR), NLRP3 inflammasome, and cGAS, also showed great superiority in enhancing the immune response to vaccines (8). TLR is one of the major pathogen recognition receptors for regulating innate and adaptive immune responses, and cognate agonists have been extensively employed as adjuvants for vaccine development (10). For example, AS04, monophosphatidyl lipid A (MPL)-containing TLR4 agonist adjuvant, was approved for the human papillomavirus (HPV) vaccine Cervarix, which showed high efficacy against cervical intraepithelial neoplasia 2+ (CIN2+) (11). The “1018 ISS adjuvant”, a synthetic nucleic acid-based CpG oligonucleotides (CpG ODNs) as TLR9 agonist, was FDA -approved for the hepatitis B vaccine Heplisav-B in 2017, which generated a potent immune response with high titers and durable antibody protection (12). Activation of the TLR2 pathway also plays an important role in tuberculosis vaccine development (13–16). In addition, several other TLR adjuvants, such as TLR3 agonist poly I: C12U, TLR5 agonist VAX102, TLR7/8 agonists Resiquimod and Imiquimod, are also undergoing clinical trials, showing enormous potential for clinical application (17–22).

Nanoparticle vaccine, as a novel and promising carrier and delivery system, can not only be used for enhancing the valence and stability of vaccines but also promote the uptake and presentation of antigens by antigen-presenting cells, thus potently regulating the immune response and improving the efficiency of vaccines (23). In the previous study, we developed a novel nanoparticle vaccine by conjugating receptor binding domain (RBD) and heptad repeats (HR) of Spike onto a Ferritin-based 24-mer particle, which elicited potent immunogenicity in rhesus macaques and protected hACE2-mice from virus infection. However, we used a mixture adjuvant purchased from Sigma company which has never been used in the clinic (24). Alternatively, the aluminum adjuvant was often used in prophylactic vaccination (25). It remains to be determined what type of adjuvants is the most suitable for self-assembled nanoparticle vaccine to enhance its efficacy.

In this study, by screening the toll-like receptor agonists, we identified that a TLR2/6 ligand analog Pam2CSK4 is the most potent adjuvant to enhance the immune response of the SARS-CoV-2 nanoparticle vaccine. We further found that Pam2CSK4 stimulates the secretion of cytokines, such as IL2 and TNF family members, and improves lymphocyte proliferation.

## Materials and methods

### Ethics statement

The Ethics Review Board of Sun Yat-sen University approved this study. Mice experiments were carried out in concert with the guidelines and regulations of the Laboratory Monitoring Committee of Guangdong Province of China. The animal experiments were also approved by the Ethics Committee of Zhongshan School of Medicine (ZSSOM) of Sun Yat-sen University on Laboratory Animal Care (Assurance Number: 2017-061). All efforts were made to avoid animal suffering.

### Cell line

HEK293T and CHO-K1 cell lines were obtained from ATCC, and the HEK293T-hACE2 cell line was established according to our previous work (24). HEK293T and HEK293T-hACE2 cell lines were cultured in Dulbecco’s modified Eagle medium (DMEM; Gibco) supplemented with 10% fetal bovine serum (FBS; Gibco) and 1% penicillin-streptomycin (Gibco) at 37°C with 5% CO2. The CHO-K1 cell line was cultured in Media C Plus (DUONING) supplemented with 1% GlutaMAX™ (Gibco) and 1% penicillin-streptomycin (Gibco) at 37°C with 5% CO2. CHO-GS-/- cell and RBD-GS-CHO cell lines were constructed by CRISPR/Cas9-mediated targeted gene integration system.

### CRISPR/Cas9-mediated targeted gene integration system

The detailed construction method of the system is in our published work (26). sgRNA sequences targeting the sixth exon of the human GLUL gene and GAPDH 3′ UTR were designed by the online tool CRISPR Design from Dr. Feng Zhang’s laboratory (https://zlab.bio/guide-design-resources). sgRNA was constructed into lentiCRISPR v2 plasmid (Addgene) with the Cas9 gene. Homologous arms with a length of 800bp were designed at both sides of the cleavage site (“GAPDH 3′ UTR”) and connected to the target fragment of gene editing, then constructed into lentiviral vector pCPPT-IRES-mStrawberry (27). Cas9/sgRNA and Target fragment with homologous arms were respectively delivered to cells *via* an integrase-deficient lentiviral vector (IDLV) for gene integration. The related sequences are in **Supplementary Table 1**.

### T7 endonuclease I assay

Cells’ genomic DNA edited by Cas9/sgRNA system was extracted and used as the template to design primers downstream and upstream of the cutting site, amplifying the mutant fragments by Polymerase Chain Reaction (PCR). The reaction system of the T7E1 digestion method: 300ng PCR product, 2 μl 10×NEB buffer2 (NEB), and ddH2O constant volume to 20 μl. It was heated at 95°C for 5 minutes and cooled naturally to room temperature. Then 0.5 μl T7E1 enzyme (NEB) was added to each reaction system and digested at 37°C for 45 minutes. Add 1 μl 0.25M EDTA stop solution to stop the reaction, and immediately detect the cutting efficiency by 1% agarose gel electrophoresis. T7E1 cleavage efficiency was analyzed and quantified using ImageJ (28). (Primer sequence in **Supplementary Table 1**)

### Cell counting kit-8 assay

Every 100 μl cell suspension was inoculated in a 96-well plate (Corning) at a density of 5×105/ml. 12 to 16 hours later, when the confluence of cells reached 70% to 80%, add 10 μl CCK-8 (DOJINDO) and incubate at 37 °C for 1 to 2 hours. measure absorption at 450 nm. Cell viability (%) = [(absorbance of experimental well - absorbance of blank well)/(absorbance of control well - absorbance of blank well)] × 100%.

### BALB/c mice experiment

Six-week-old female BALB/c mice were purchased from Guangdong Medical Laboratory Animal Center. All mice were housed and vaccinated in Specific-pathogen-free (SPF) facilities at the Laboratory Animal Center of Sun Yat-sen University. The RBD-nanoparticle (RBD-NP) vaccine was constructed and purified as previously described (24). Briefly, we purified and conjugated SARS-CoV-2 spike protein receptor binding domain (RBD) (Genbank: QHD43416.1) to 24-mer Ferritin (UniProt: Q9ZLI1) nanoparticles by SpyTag/SpyCatcher (PDB: 4MLI) (ST/SC) system. ST-RBD protein purified from RBD-GS-CHO cells and SC-Ferritin core purified from *E.coli* was covalently bound in a molar ratio of 6:5 and then collected by Size-Exclusion Chromatography (SEC). The concentration was measured by BCA assay, and the purity and homogeneity of RBD-NP were determined by Coomassie blue staining, western blotting, SEC, and transmission electron microscopy (TEM). Mice were immunized subcutaneously with 5.6 ug RBD-NP vaccine alone or in combination with different adjuvants at week 0. Mice were immunized again at week 4. The dosage of various adjuvants was different (**Supplementary Table 2**). All mice were euthanized at week 10.

### Enzyme-linked immunosorbent assay

Recombinant ST-RBD protein at a concentration of 2 ug/ml in coating buffer was coated on high-binding 96-well plates (Corning) respectively, overnight at 4°C. After coating, drain the liquid and block the plates with 5% non-fat milk/PBS at room temperature for 1 hour. Diluted Immunized mice serum into eight different concentrations with PBS at a 3-fold concentration gradient and added them into the well plates each well in duplicate followed by incubating at room temperature for 1 hour. After washing with PBS/T (containing 1% Tween-20), the detection of RBD-specific IgG and RBD-specific IgM in the serum of BALB/c mice was conducted by adding HRP-conjugated goat anti-mouse secondary antibody (Invitrogen) at dilution of 1:6000 and incubating at 37°C for 1 hour. After washing with PBS/T, add HRP substrate TMB solution (eBioscience) to each well, quench reaction with stop solution (Solarbio), and measure absorption at 450 nm.

### Enzyme-linked immune absorbent spot assay

The detailed protocol was developed by following previous procedures with minor modifications (29). Briefly, multiScreen_HTS_ IP filter plates of 0.45 μm (Millipore Sigma) were coated with 2.5 mg/mL SARS-CoV-2 RBD protein at 4 °C for 12 hours. Then plates were washed three times with PBS and blocked with DMEM at 37 °C for 1 hour. One million mice bone marrow cells were harvested and incubated inside each well at 37 °C overnight. After washing with PBS/T three times, plates were incubated with anti-IgG-HRP antibody (Jackson ImmunoResearch) at room temperature for 2 hours. Plates were washed with PBS/T three times, RBD-specific spots were then counted using an S6 ultra immunoscan reader (Cellular Technology Ltd.).

### Pseudotyped virus neutralization assay

The serum neutralizing antibody titers in immunized mice were measured as previously described. Briefly, pseudotyped SARS-CoV-2 viruses were obtained by co-transfection of packaging plasmid psPAX2 (Addgene), luciferase-expressing lentivirus plasmid pHIV-Luciferase (Addgene) and plasmid expressing SARS-CoV-2 spike protein (isolate Wuhan-Hu-1, GenBank: QHD43416.1) into HEK293T cells. Then serially diluted serum of BALB/c mice were mixed with pseudotyped SARS-CoV-2 viruses and incubated at 37°C for 1 hour. Serum/virus mixtures were added into wells which were seeded with hACE2-HEK293T cells and went on culturing for 48 h. After 48 h, cells were lysed with lysis buffer (Promega) and were measured for relative luminescence units in luminometer (Promega).

### Flow cytometry and intracellular cytokine staining

The cell phenotypes were analyzed by FACS Aria II flow cytometer (BD Bioscience), and data were analyzed with the FlowJo V10.0.7 (FlowJo, OR, USA). Cells were collected and washed in PBS, then the single-cell suspensions were labeled with various fluorochrome-conjugated antibodies for 30 minutes within PBS containing 0.5% BSA on ice. The gating strategies for CD4+ central memory T cells, CD8+ central memory T cells, CD4+ effector memory T cells, Tfh cells were CD4+CD62L+CD44+, CD8+CD62L+CD44+, CD4+CD62L-CD44+, CD4+CXCR5+PD-1+, respectively. The following antibodies were used: anti-mouse CD4-AF700 (clone RM4-5, eBioscience), anti-mouse CD8a-BV510 (clone 53-6.7, BD Horizon), anti-mouse CD62L-PE (clone MEL-14, Biolegend), anti-mouse CD44-Percp/Cy5.5 (clone 1M7, Biolegend), anti-mouse CXCR5-APC (clone SPRCL5, eBioscience) and anti-mouse PD-1-PE/Cy7 (clone J43, eBioscience). For ICCS, mice splenocytes were collected and co-stimulated with anti-CD3 antibody and anti-CD28 antibody (Biolegend) for 1 hour at 37 °C, then Cells were incubated with 5 mg/mL brefeldin A (Topscience), 2 mM monensin (Topscience). Cells were then harvested, stained with indicated antibodies with a Fixation/Permeabilization Solution Kit (BD Bioscience). We used these antibodies to detect cytokine secretion of T cells: anti-IFN-γ (clone XMG1.2, Biolegend) and anti-IL-2 (clone JES6-5H4, Biolegend).

### RNA-sequencing and transcriptional profiling

BALB/c mice were injected with SARS-CoV-2 RBD Nanoparticle vaccine with or without Pam2CSK4. The peripheral blood mononuclear cells were isolated with the Ficoll method on day 3 and day 7 after the immunization as previously [Luo, B et al., 2020 CDD]. For RNA-sequencing analysis, total RNAs were extracted by TRIzol Reagent (Thermo Fisher Scientific) according to the manufacturer’s instructions. The quality of RNA samples was evaluated by Nanodrop 2000 (Thermo Fisher). The RNA-Seq library was built with TruSeq Stranded mRNA Library Prep Kit (Illumina) and sequenced with Hiseq X Ten (Illumina) at BioMarker (Beijing, China) under the PE150 protocol. RNA-Seq reads were trimmed, filtered, and quality-controlled by the FastQC tool. Followed by calculating the reads per kilobase per million mapped reads (RPKM), the reads were aligned with human reference genome NCBI build 38 (GRC38) by Hisat268. The cutoff of fold-change (FC) ≥2 and FDR values <0.05 was used as the criterion. Through z-score normalization, the transcriptional profile data of differentially expressed genes were presented in a heatmap with MEV software.

### Statistical analysis

Experiments were conducted independently at least three times, and mice were tested with at least five mice in each group. Software GraphPad Prism 8.0 and OriginPro 8.0 (OriginLab) were used to process and analyze the data, and data were represented as mean ± SEM. Two-tailed student’s t-test was used to compare Two independent groups, and one-way ANOVA was used in Tukey’s multiple comparisons test. Data are considered statistically significant when *p* < 0.05.

## Results

### Generation of SARS-CoV-2 RBD immunogen from CRISPR/Cas9-engineered CHO-K1 cells

**Figure 1:**
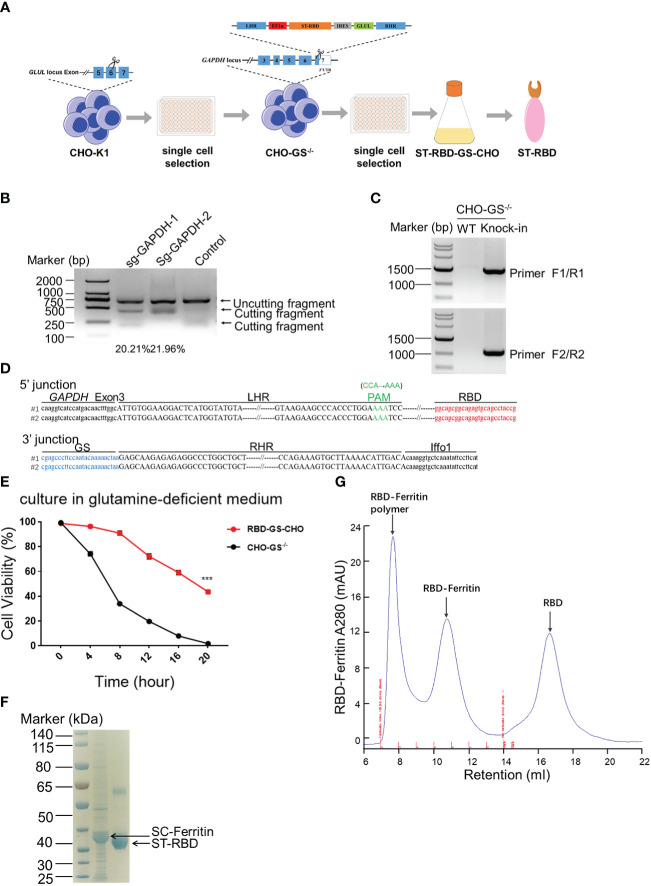
Construction of CHO-GS cells stably expressing RBD protein using CRISPR/Cas9 system for the assembly of the SARS-CoV-2 RBD NP Vaccine. **(A)** ST-RBD-GS-CHO cell construction strategy: CHO-K1 cells were transfected with Cas9/sgRNA targeting the GLUL gene, and 24 hours later, CHO-GS-/- cells were screened with puromycin for 3 days and subjected to single-cell screening with amplification. The ST-RBD-GS gene was then knocked into the GAPDH locus by the CRISPR/Cas9 system to consecutively express the RBD protein. **(B)** CHO-GS-/- cells were transfected with Cas9/sgRNA and donor DNA targeting the GAPDH gene by IDLV infection, and the editing efficiency of sgRNA was detected by the T7E1 assay as previously. Negative control was an unrelated sgRNAs. **(C, D)** Single cells were seeded and amplified, and then genomic DNA was extracted and amplified by PCR using primers F1/R1 and F2/R2, primer sites were shown in **Figure 1A**, respectively. The negative control was CHO-GS-/- cells. C: PCR amplification of fragments to confirm the insertion; D: blast results for DNA sequencing. **(E)**. Cell viability of CHO-GS-/- and RBD-GS-CHO cells was determined by CCK-8 assay in the glutamine-free medium. **(F)** 10 μg of purified ST-RBD and SC-ferritin samples were analyzed by SDS-PAGE electrophoresis and stained with Coomassie Blue. **(G)** Purification and concentration of RBD-Ferritin nanoparticles by size exclusion chromatography. The UV absorption value at 280 nm indicates the abundance of protein molecules. The X-axis indicated the retention volume. All the experiments were performed at least 3 times independently.

Chinese Hamster Ovary (CHO) cells are widely used for protein expression and production in eukaryotic systems in biopharmaceuticals (30, 31). Traditional CHO cell production pipeline is always heavy workload and time-consuming. However, a glutamine synthetase (GS) system was introduced into the CHO cell platform for skipping the amplification before screening and enhancing yielding with fewer gene copies to integration (32). Therefore, we intended to construct a CHO-GS cell line by inserting ST(SpyTag)-RBD-GS at the GAPDH site for steadily producing RBD protein (**Figure 1A**). To this end, we first constructed the CHO-GS cell line to abolish the function of GLUL in CHO-K1 cells, which encoded glutamine synthetase (**Figure 1A**). Four sgRNA candidates were designed on both sides of exon 6 of the GLUL gene, followed by co-transfection with a Cas9-expressing plasmid. The editing efficiencies of these sg-GLUL RNAs were determined by the T7E1 assay, which could recognize mutations resulting from repair after Cas9 cutting (**Supplementary Figure 1A**). The sg-GLUL-1 and sg-GLUL-3 were chosen for subsequent knock-out editing for their high efficiency (**Supplementary Figure 1A**). The knockdown of the double allele in monoclonal CHO-GS-/- cells was also confirmed by DNA sequencing. Furthermore, CHO-GS-/- cells cannot survive in a glutamine-deficient medium to verify the inactivation of glutamine synthetase by CCK-8 assay (**Supplementary Figure 1B**). Taken together, the CHO-GS-/- cells were successfully constructed and confirmed from multiple aspects, which could be used for subsequent screening experiments.

We then constructed a plasmid expressing ST-RBD-GS, which is the receptor-binding domain (RBD) immunogen of the SARS-CoV-2 original strain connected with an ST-tag, leading to its covalent conjugation onto the nanoparticle surface *via* the interaction between SpyTag and SpyCatcher (24). The 3’ UTR region of the GAPDH was chosen for ST-RBD-GS insertion, which allowed high-yield expression of exogenous proteins, as described in our previous work (26). The homologous arms of LHR and RHR of the target locus were put on both sides of ST-RBD-GS, which would be integrated into the CRISPR/Cas9-mediated cleavage site by homologous recombination (**Figure 1A**). The sgRNAs candidates were also screened by the T7E1 assay as previously, in which sg-GAPDH-2 was a high editing effect, and it was selected to assist the insertion of ST-RBD-GS (**Figure 1B**). The site-specific integration of ST-RBD-GS was further confirmed by sequencing (**Figures 1C** **, D**). The resulting CHO cell line, named RBD-GS-CHO cells, grew much better in the glutamine-free medium than CHO-GS-/- cells, verifying the successfully constructed screening system and the right cell line with anaplerosis of GS (**Figure 1E**). Furthermore, the expression and purification of ST-RBD in the medium were confirmed by the analysis of SDS-PAGE electrophoresis (**Figure 1F**). To assemble the SARS-CoV-2 nanoparticle vaccine, the SC-Ferritin fusion protein was effectively purified from E.coli as previously (**Figure 1F**) (24). Protein expression, purification, assembly, and molecular sieve enrichment were performed sequentially to obtain RBD-nanoparticle (RBD-NP) vaccines (**Figure 1G** and **Supplementary Figure 2**).

### The preliminary screening of the TLR agonists-adjuvanted SARS-CoV-2 RBD nanoparticle vaccine on the humoral immune response

**Figure 2:**
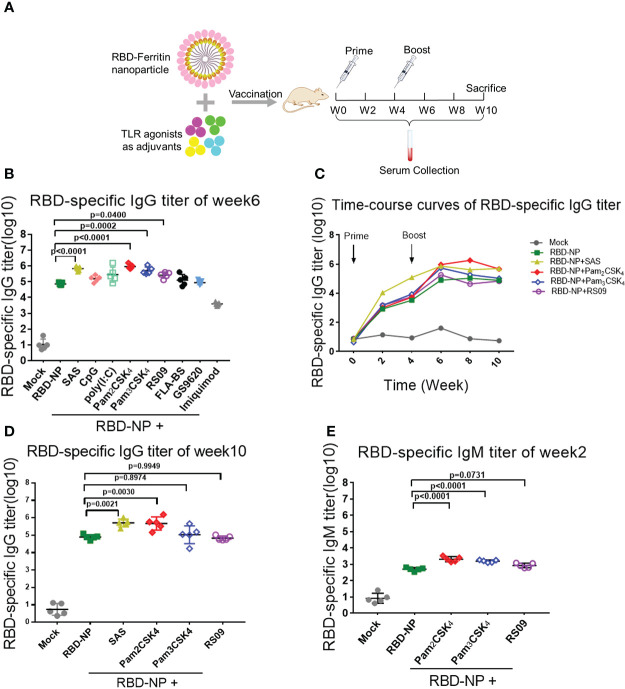
Screening of TLR agonist adjuvant to enhance humoral immune response elicited by SARS-CoV-2 nanoparticle vaccine. **(A)** Schematic design of BALB/c mice immunized with the Prime-Boost strategy. Sera were collected every two weeks after the initial immunization. **(B–D)** Monitor SARS-CoV-2 RBD-specific IgG titers in immunized mice by ELISA from week 0 to week 10. IgG titers of sera were measured by serial dilution and represented as the reciprocal of the endpoint serum dilution (n = 5). B: IgG titers of sera at week 6; c: RBD-specific IgG titers were calculated every two weeks and plotted as a time-course curve; D: IgG titers of sera at week 10. **(E)** RBD-specific IgM titers of immunized mice were collected by ELISA at week 2 (n = 5). One-way ANOVA was used in Tukey’s multiple comparison test. Experimental data are expressed as Mean ± SEM.

To search for the optimal adjuvants for SARS-CoV-2 RBD-NP vaccines, nine adjuvant candidates that are TLR agonists used in clinical trials were respectively used in combination with the RBD-NP vaccines (**Supplementary Table 2**). The Sigma Adjuvant System (SAS), although not used in clinical trials so far, served as the positive control to compare the immune response. The prime-boost strategy was implemented to elicit the immune response in the BALB/c mice, which were vaccinated at week 0 and week 4 (**Figure 2A**). To evaluate the humoral immunity induced by the adjuvanted RBD-NP vaccine, we monitored the titers of RBD-specific IgG by ELISA at multiple time points. After boost immunization, the IgG antibodies in the groups of Pam2CSK4, Pam3CSK4, and RS09 were significantly elevated more than that in other adjuvanted groups (**Figure 2B**). The Pam2CSK4-adjuvanted groups produced approximately ten times higher IgG titers than those in the non-adjuvant group after boost immunization (*p*<0.0001) (**Figure 2B**). Furthermore, the IgG titers in the Pam2CSK4, Pam3CSK4, and RS09 groups were effectively maintained at higher levels for a long time (**Figures 2C** **, D**). Long-lived plasma cells (LLPCs) could produce specific antibodies for a long time, which play key role in eliciting durable humoral responses (33). In our study, the Pam2CSK4 group induced more LLPCs producing RBD-specific antibody than the other groups by Enzyme-Linked Immune Absorbent Spot (ELISpot) (**Supplementary Figure 3**). Since IgM is the earliest type of antibody produced after infection or vaccination, we examined the titers of RBD-specific IgM in the Pam2CSK4, Pam3CSK4, and RS09 groups and found that they were much higher than the non-adjuvant group in the early time (**Figure 2E**). The above data suggest that these three adjuvants could potently boost and maintain higher levels of protective antibodies including both IgG and IgM.

**Figure 3:**
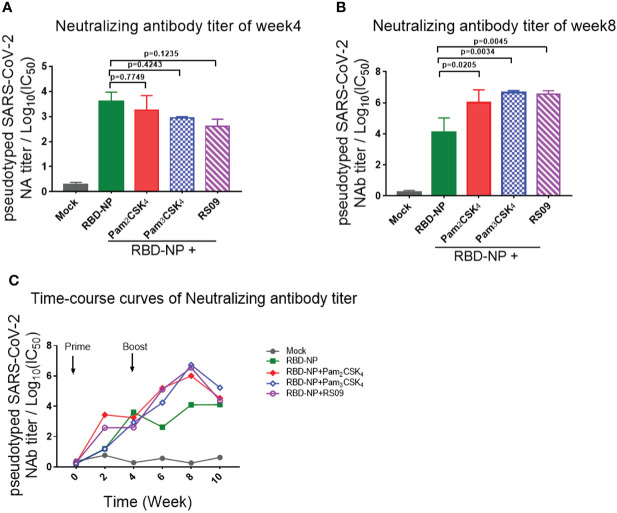
Detection and comparison of the neutralizing antibodies titer elicited by TLR agonist-adjuvanted SARS-CoV-2 nanoparticle vaccine. **(A–C)** Mice were immunized with the Prime/Boost strategy. Neutralizing antibody titers were detected by pseudotyped SARS-CoV-2 virus neutralization assay, which reflected the capability of antibodies to block the virus entry (n=5). Log_10_(IC50) indicates neutralizing antibody titers. **(A)** Time course curve from week 0 to week 10; **(B)** Neutralizing antibody titer at week 4; **(C)** Neutralizing antibody titer at week 8. Experiments were performed independently at least 3 times. One-way ANOVA was used in Tukey’s multiple comparison test. Experimental data are expressed as Mean ± SEM.

To further determine whether these adjuvants could improve the generation of neutralizing antibodies elicited by the RBD-NP vaccine, which reflected the capability of the antibodies to block the virus entry through the interaction between Spike and ACE2, we constructed a SARS-CoV-2 Spike protein-expressing plasmid for packaging the pseudotyped SARS-CoV-2 S/HIV-1 virus as described previously (24). The luciferase gene was inserted into the HIV-1 vector and can be expressed after infection with the pseudotyped viruses. We incubated the sera with pseudotyped SARS-CoV-2 S/HIV-1 virus, followed by the infection of HEK293T-ACE2 cells and the detection of luciferase. The results showed that neutralizing antibodies were produced at week 4 after prime immunization, without any differences between the adjuvant and control groups (**Figure 3A**). However, the titers of neutralizing antibodies in mice peaked at week 4 after boost immunization. Compared to the non-adjuvant group, the Pam2CSK4, Pam3CSK4, and RS09 groups produced approximately 70-fold higher neutralizing antibodies titers than the non-adjuvant group (**Figures 2B** **, C**). Taken together, the adjuvants, including Pam2CSK4, Pam3CSK4, and RS09 can contribute to a higher protective humoral immune response to the RBD-NP vaccine by eliciting higher titers of serum neutralizing antibodies, RBD-specific IgM, and IgG. Importantly, Pam2CSK4 is superior to the other two adjuvants in helping to maintain high IgG potency, leading to substantial durability of antibody protection.

### Evaluate the cellular immune responses of the TLR agonists-adjuvanted SARS-CoV-2 RBD nanoparticle vaccine

**Figure 4:**
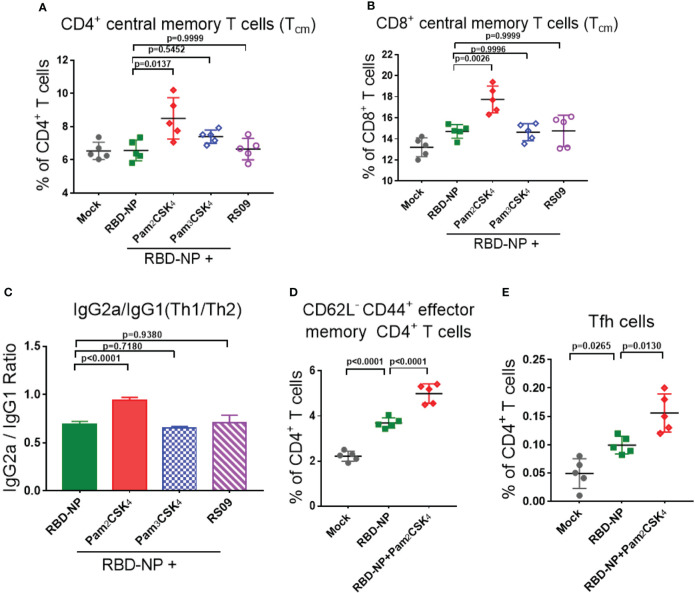
Evaluate the cellular immune responses of the adjuvanted SARS-CoV-2 nanoparticle vaccine *in vivo*. **(A, B)** Splenocytes were collected at week 10 and the central memory T cells were detected by flow cytometry. **(A)** CD4+CD62L+CD44+ Tcm cells; **(B)** CD8+CD62L+CD44+ Tcm cells (n=5). **(C)** IgG2a and IgG1 titers in sera were measured by ELISA at week 6, and the ratio of IgG2a/IgG1 represented a trend of Th1/Th2 response (n=5). **(D)** Mandibular lymph nodes were collected and the central memory CD4+ T cells (CD4+CD62L-CD44+ T cells) were analyzed 10 days after immunization (n=5). **(E)** Tfh cells (CD4+CXCR5+PD-1+) were collected from lymph nodes and analyzed 10 days after immunization (n=5). Experiments were performed at least three times independently. One-way ANOVA was used for Tukey’s multiple comparison test. Experimental data are expressed as Mean ± SEM.

To explore whether these three adjuvants could also enhance the cellular immune responses, we analyzed the percentage of central memory T cells (Tcm) in the spleen of mice by flow cytometry ten weeks after the initial vaccination. Pam2CSK4 significantly promoted the production of more CD4+Tcm cells (CD4+CD62L+CD44+T cells) and CD8+Tcm cells (CD8+CD62L+CD44+T cells) than Pam3CSK4 and RS09, indicating a potential long-term protection from the immune memory (**Figures 4A** **, B**). Moreover, T cells in Pam2CSK4-adjuvanted group were more responsive to the re-stimulation ten weeks post prime vaccination, in accordance with the more expression of the IFN-γ and IL-2 (**Supplementary Figure 4**).

Safety is as equally important as efficacy in the vaccine and adjuvant development. The type 2 T helper (Th2)-biased immune response is potentially associated with vaccine-associated enhanced respiratory disease (VAERD) and hypersensitivity (34, 35). In addition, it was reported that the Th1-biased immune response could enhance the resistance to SARS-CoV-2 infection (35, 36). In the mouse model, the IgG2a/IgG1 ratio was usually used to indicate the type bias of the Th1/Th2 response, in which IgG2a had a high correlation with the Th1-biased immune response. Through analyzing the antibodies in the sera of mice 6 weeks after initial immunization, only Pam2CSK4 could induce Th1-type immune responses, indicating superior and safer protection compare to another two adjuvants (**Figure 4C**, **Supplementary Figure 5**). The above data suggested that Pam2CSK4 has a positive effect on improving the efficacy and safety of the immune response induced by the RBD-NP vaccine.

To further investigate whether Pam2CSK4 could promote the differentiation of fast-acting and protective effector memory T cells (Tem), lymph node cells were extracted from mice 10 days after vaccine immunization. Compared to the non-adjuvanted group, there were more antigen-stimulated CD4+Tem cells (CD4+CD62L-CD44+ T cells) in the Pam2CSK4-adjuvanted group, indicating a boosting role of Pam2CSK4 in antigen presentation and T cell activation (**Figure 4D**). In addition, it was reported that nanoparticle vaccines were more readily captured and presented by APC, which facilitated follicular helper T (Tfh) cells to participate in the synergistic regulation between T cells and B cells (37, 38). Indeed, Pam2CSK4 significantly increased the Tfh cells in mouse lymph nodes ten days after immunization (**Figure 4E**). Taken together, our results suggested that Pam2CSK4 enhanced the immune response elicited by the RBD-NP vaccine, by promoting protective Th1-biased reaction, improving antigen presentation, and facilitating both memory and follicular T cell differentiation.

### The molecular signatures of Pam2CSK4-adjuvanted SARS-CoV-2 nanoparticle vaccine

**Figure 5:**
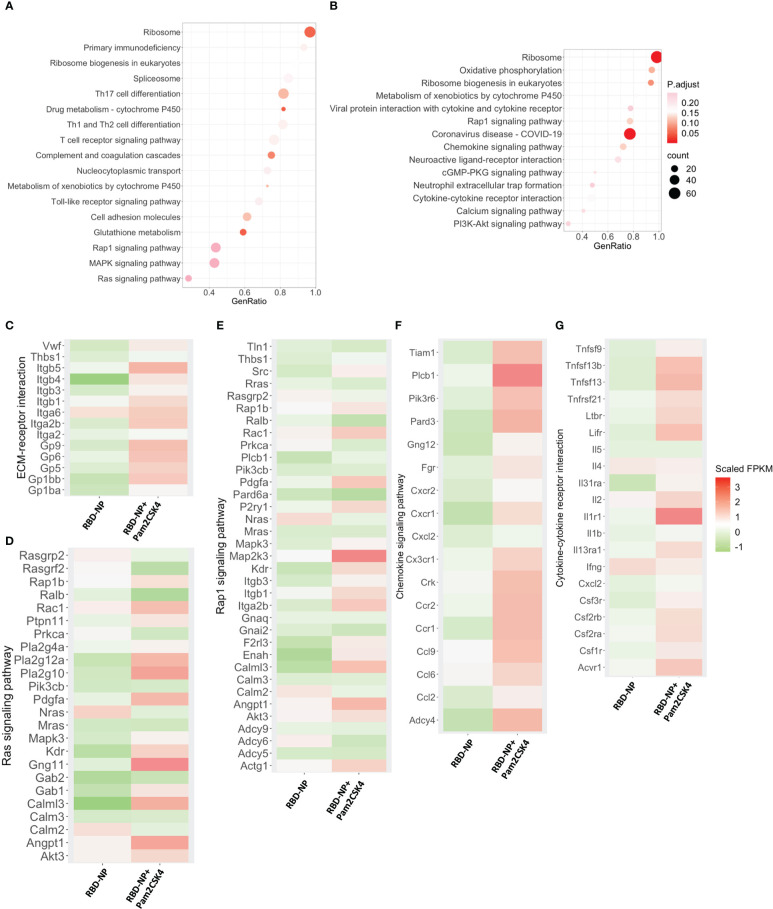
Transcriptional characterization of Pam2CSK4-adjuvanted SARS-CoV-2 nanoparticle vaccine. **(A, B)** Major gene enrichment maps of GSEA for peripheral leukocytes in the RBD-NP and RBD-NP+Pam2CSK4 groups of mice, respectively. Circles represent the number of genes involved in the gene pathway. **(A)** three days after immunization; **(B)** 7 days after immunization. **(C–E)**. Heat map of genes associated with ECM-receptor interactions, Ras, and Rap1 signaling pathways showing the activation enrichment in the Pam2CSK4 + RBD-NP group compared to the RBD-NP group at day 3 post-immunization. **(F, G)** A heat map of genes associated with chemokine signaling and cytokine-cytokine receptor interactions pathways shows activation enrichment in the Pam2CSK4 + RBD-NP group compared to the RBD-NP group at day 7 post-immunization. Color changes represent Scaled FPKM. RNA expression levels are indicated by red/green Scaled FPKM for high and low expression levels, respectively.

Upon immunization, the immune system responds with changes associated with cell activation and the coordination of various cellular subpopulations and pathways (39). To further explore the regulatory mechanisms of how Pam2CSK4 enhanced the protective immune response of the RBD-NP vaccine against SARS-CoV-2, we compared the molecular characteristics of peripheral blood leukocytes in the Pam2CSK4-adjuvanted RBD-NP group with those in the non-adjuvant group. Three days after vaccination, gene set enrichment analysis (GSEA) showed that Pam2CSK4 promoted the SARS-CoV-2 nanoparticle vaccine with significant changes in ribosomal and cell adhesion pathways, consistent with cell activation and migration. Moreover, the metabolic levels of the immune system were significantly elevated to provide energy for immune response at different time points, such as the glutathione metabolic pathway (**Figures 5A, B**, **Supplementary Figure 6**). Meanwhile, the transcriptional analysis revealed significant upregulation of genes involved in extracellular matrix (ECM)-receptor interactions and RAS signaling pathways, such as *Itgb5*, *Itga6*, *Calml3*, and *Gp6*, in Pam2CSK4-adjuvanted SARS-CoV-2 nanoparticles (**Figures 5C** **–E**). Compared to the non-adjuvant group, these genes were more involved in leukocyte adhesion in the Pam2CSK4 group. Furthermore, the transcriptional profile also showed Pam2CSK4 promoted RAP1 signaling pathways activation, including *Mapk3*, *Rac1*, *Pdgfa*, and *Gng11* (**Figures 5D** **, E**). Taken together, our results suggested that Pam2CSK4 can effectively promote cell differentiation and proliferation through multiple cell activation-associated pathways.

GSEA was also carried out for comparing the differences on day 7 after vaccination between Pam2CSK4-adjuvanted and non-adjuvanted groups. Pam2CSK4 increased more expression of genes associated with chemokine signaling and cytokine-cytokine receptor for the SARS-CoV-2 NP vaccine (**Figure 5B**). The upregulation of related genes involved mediates leukocyte activation and leukocyte migration, including *Il2*, *Ccl2*, *Ccl6*, *Ccl9*, *Cxcl2*, *Cx3cr1*, *Il1r1*, *Cxcr1*, *Ccr1*, *Ccr2*, and importantly, *Tnfsf13* (*April*) (**Figures 5F** **, G**). Remarkably, the upregulation of *Il2*, *Ccl2*, *Csf3r*, and the downregulation of *Il4* were consistent with the Th-1-biased immune response, further suggesting that Pam2CSK4 could induce a potent and safe immune response (**Figures 5G** and **4C**) (40). Overall, the transcriptional profiling analysis revealed a positive molecular signature induced by Pam2CSK4-adjuvanted SARS-CoV-2 nanoparticle vaccination.

## Discussion

The massive requirement for vaccines against the COVID-19 pandemic has also driven the urgent demand for adjuvant development (41). Based on previous work, CHO cells were genetically edited by the CRISPR/Cas9 system to efficiently and continuously secrete RBD protein, facilitating the production of the nanoparticle vaccine in the clinical standard. Meanwhile, by screening and comparing all TLR agonists in clinical trials, we found that Pam2CSK4 can significantly enhance both humoral and cellular immune responses of RBD-based nanoparticle vaccines. Furthermore, Pam2CSK4 induced a much safer and more potent Th1-bias reaction and showed advantages in maintaining immune memory and T-B cell coordination, which was also demonstrated by the transcriptional profiles of mouse peripheral leukocytes. Taken together, Pam2CSK4, targeting TLR2/6, is a potent adjuvant that can enhance the efficacy of SARS-CoV-2 nanoparticle vaccines by boosting the protective immune response.

The launch of mRNA-1273 and NVX-CoV2373 has drawn attention to nanoparticle vaccines (42–44). The RBD-Ferritin nanoparticle vaccine developed in our early stage has played an excellent advantage with the assistance of the SAS (24). To further promote the clinical translation of this nanoparticle vaccine, we used the CHO system commonly used in clinical biological products to further optimize it. There are three advantages to employing the CHO system for RBD protein preparation. First, CHO cells could endow the Spike/RBD with the natural folding confirmation and glycosylation modification compared to the prokaryotic bacterial system. Second, the CHO system has been a proven production standard for the pharmaceutical industry and is widely applied in protein production. Third, the CHO system could produce large-scale protein in a high density in the serum-free medium (31, 45, 46). In combination with the site-specific genetically engineered CRISPR/Cas9 technology, the CHO system got further improvement in efficiency and standardization (47).

In addition, adjuvants played an essential role in improving immunogenicity and boosting efficacies and we tried to find a clinically relevant adjuvant match for this nanoparticle vaccine. Since alum adjuvant was first shown to improve diphtheria vaccine protection in 1926, it has become the most commonly used adjuvant to persistently activate the adaptive immune response by the slow release of antigens and the prolonged interaction with APC (48, 49). Other emulsion adjuvants, such as AS01, AS03, and MF59, have also been shown to enhance vaccine immune response *via* the mechanisms that induce cell immigration and improve APC activation by increasing the secretion of related cytokines (50–52). NVX-CoV2373, a recent FDS-approved SARS-CoV-2 nanoparticle vaccine, is composed of a trimeric full-length Spike protein, adjuvanted with Matrix-M1, a saponin-based compound (42, 53). It remains to be elucidated the combination principle for the vaccines and various adjuvants. Along with the in-depth understanding of the immune regulation post-vaccination, it has been found that the recognition and activation mediated by pattern recognition receptors (PRRs) are essential to bridge the innate immune to adaptive immune. Because TLR agonists have been widely used in vaccine studies and showed extensive safety and efficacy, we performed the screening of all the TLR agonists to look for a potent adjuvant to boost the immune response of the SARS-CoV-2 nanoparticle vaccine. We demonstrated that TLR2/6-targeted Pam2CSK4 played a potent role in inducing high and more durable titers of antibody production than any other TLR agonist (**Figure 2**). Pam2CSK4 also accelerated the immune reaction to produce a protective effect *in vivo*, showing its superiority (**Figure 3**).

It’s reported that there was a significant decrease in serum neutralizing antibody levels after recovery in some COVID-19 patients (54, 55). Therefore, it is remarkable that Pam2CSK4 can also play a key role in long-term protection by inducing more memory T cells and Tfh cells (**Figure 4**). Furthermore, molecular signatures of transcriptional profiles showed the pathways related to cell migration, activation, proliferation, and adhesion were most activated during immunization of Pam2CSK4-adjuvanted nanoparticles vaccine, contributing to the potent and long-lasting immune protection (39, 56). Importantly, the upregulated expression of *APRIL*, which is a key ligand for the differentiation of long-lived plasma cells, could lead to a durable production of nAbs against SARS-CoV-2 (57–59). Together, we provide an important theoretical principle of the combination of SARS-CoV-2 NP vaccine and adjuvant for clinical transformation.

The safety of the adjuvants is the priority for vaccine development. It has been shown that Pam2CSK4 is a Th2 polarizing adjuvant in Chlamydia vaccine, Leishmania major and Brugia malayi murine vaccine in clinical trials (60, 61). However, other studies have shown that TLR2 agonist-adjuvanted tuberculosis vaccine can induce a Th1-biased immune response (13, 16). Our data indicate that the Pam2CSK4-adjuvanted SARS-CoV-2 NP vaccine induced a Th1-biased immune response in our results (**Figure 4C**), which was also confirmed by the analysis of transcriptional profiling that *IL2* and *TNF* family genes were significantly elevated in the Pam2CSK4-adjuvanted group (**Figure 5G**). In addition, previous studies on SARS-CoV believed that a Th1-biased immune response is also considered as much safer than a Th2-biased one, reducing the risk of VAERD and contributing to the safety assessment (34, 35).

In summary, we engineeringly modified the RBD preparation to produce the SARS-CoV-2 nanoparticle vaccine, paving the way for rapid transformation. We also demonstrated that Pam2CSK4, a TLR2/6 agonist already used in clinical trials with other vaccines, was the most potent adjuvant to boost the immune response of the RBD-NP vaccine. This adjuvanted-nanoparticle vaccine has the potential advantages of high antibody titers, durable efficacy, and safety for further clinical use to prevent COVID19.

## Data availability statement

The datasets presented in this study can be found in online repositories. The names of the repository/repositories and accession number(s) can be found below: All RNA-seq data have been deposited in the Sequence Read Archive (SRA) under accession number PRJNA857815.

## Ethics statement

The animal study was reviewed and approved by the Ethics Committee of Zhongshan School of Medicine (ZSSOM) of Sun Yat-sen University.

## Author contributions

Conceptualization, HZ and XH. Methodology, YQ, YKZ, YLZ and JD. Investigation, BL, AC, YWZ, TP and WZ. Writing – original draft, YQ and YKZ. Writing – Review & editing, HZ and XH. Supervision, HZ and XH. All authors contributed to the article and approved the submitted version.
