# Supplementary material for: Pam2CSK4-adjuvanted SARS-CoV-2 RBD nanoparticle vaccine induces robust humoral and cellular immune responses
Source: Front Immunol. 2022 Dec 9;13:992062. doi: 10.3389/fimmu.2022.992062 (PMC9780597; doi:10.3389/fimmu.2022.992062)

Pam2CSK4-adjuvanted SARS-CoV-2 RBD Nanoparticle Vaccine induces robust humoral and cellular immune responses

Yidan Qiao[1, †], Yikang Zhan[1, †], Yongli Zhang[1], Jieyi Deng[1], Achun Chen[1], Bingfeng Liu[1], Yiwen Zhang[1], Ting Pan[1,2], Wangjian Zhang[3], Hui Zhang[1,4, *] and Xin He[1, *]

[1] Institute of Human Virology, Department of Pathogen Biology and Biosecurity, and Key Laboratory of Tropical Disease Control of Ministry of Education, Zhongshan School of Medicine, Sun Yat-sen University, Guangzhou 510080, China

[2]Center for Infection and Immunity Study, School of Medicine, Sun Yat-sen University, Shenzhen, Guangdong, 518107, China

[3]Department of Medical Statistics, School of Public Health, Sun Yat-sen University, Guangzhou, Guangdong, 510080, China

[4]Guangzhou National Laboratory, Bio-Island, Guangzhou, Guangdong, 510320, China

[†]These authors contributed equally

*Corresponding authors:

Xin He; E-mail: hexin59@mail.sysu.edu.cn ;

Hui Zhang; E-mail: zhangh92@mail.sysu.edu.cn

Supplementary Table

Supplementary Table 1 SgRNA and primer sequences

Gene	Target Sequences	PAM
sgRNA of GLUL		
Sg- GLUL -1	CATGGCCTTGGTGCTAAAGT	TGG
Sg- GLUL -2	CCTTGGTGCTAAAGTTGGTA	TGG
Sg- GLUL -3	CCAGTTCCCAGGAATGGGCT	TGG
Sg- GLUL -4	CCAAGCCCATTCCTGGGAAC	TGG
sgRNA of GAPDH		
Sg- GAPDH -1	TGCTCGAGTCCTTGCTGGGG	TGG
Sg- GAPDH -2	CGAGTCCTTGCTGGGGTGGA	TGG
Primer of integration site		
F1	CCACCAACTGCTTAGCCCCCCTG	
R1	GTCCCTCTCGAATGGCTTGAGGTTAG	
F2	GGAGGCCATCGAGAAACTAAGCAAG	
R2	GCTGACAGTGGAATCTCTAAAAGCAATG	
Primer of T7E1 Assay		
GLUL_T7E1-F	GTAAGTAGAACAAGCTAGGAGCTTGAG	
GLUL_T7E1-R	GAGGGTCTGACCTTGTCACTAGAAACC	
GAPDH_T7E1-F	CTGGAGAAACCTGTATGTCTGGGG	
GAPDH_T7E1-R	ACCTTGCTAGGTCACAGCCTCCCTC	

Supplementary Table 2 TLR agonists as adjuvants in clinical trials and vaccine studies

Adjuvant	Targeting-TLR	vaccine application	the dosage per mouse
Poly I:C	TLR3	Rabies, Influenza vaccines	100 ug
Flagellin (FLA-BS)	TLR5	Quadrivalent Influenza vaccine VAX2021Q	7.5 ug
Imiquimod	TLR7	Influenza, Hepatitis B, Varicella zoster	100 ug
Vesatolimod (GS9620)	TLR7	Hepatitis B	30 ug
CpG ODN	TLR9	Hepatitis B, SARS-CoV-2	30 ug
Pam_2CSK_4	TLR2/6	Leishmania, Brugia malayi murine vaccines	7.5 ug
Pam_3CSK_4	TLR1/2	Influenza subunit vaccine, Leishmania	15 ug
RS09	TLR4	HIV-1	30 ug

Supplementary Figure Legends

Supplementary Figure 1 Construction of CHO-GS cells by using CRISPR/Cas9 system

(A) Four candidate sgRNAs targeting the exon 6 locus of the GLUL gene were transferred into CHO-K1 cells. Genomic DNA was extracted and the editing efficiency of sgRNAs was analyzed by the T7E1 assay. T7E1 cleavage efficiency was analyzed and quantified using Image J [50]. Negative controls were unrelated sgRNAs. (B) CHO-K1 and CHO-GS-/- cells were cultured in glutamine-free medium, respectively. Cell viability was detected by CCK-8 assay.

Supplementary Figure 2 Protein purification and construction of RBD-Ferritin nanoparticle vaccine

(A) ST-RBD and SC-Ferritin were expressed by E. coli and RBD-GS-CHO cells, the two proteins were purified by His-tag affinity chromatography in Tris-NaCl buffer system.

Supplementary Figure 3 Detection of the RBD-specific long-lived plasma cells (LLPCs) elicited by Pam2CSK4-adjuvanted SARS-CoV-2 nanoparticle vaccine

(A) Mice bone marrow cells were collected at week 10 and the RBD-specific LLPCs were detected by enzyme-linked immune absorbent spot. One million cells were seeded for each well and the LLPC-secreted antibodies were captured by the RBD-coated plate.

Supplementary Figure 4 Evaluate the cellular immune responses of the adjuvanted SARS-CoV-2 nanoparticle vaccine in vivo

(A and B) Splenocytes were collected at week 10 and the cytokines secretion of CD4+T cells were detected by intracellular cytokine staining. A: IFN-γ secreted by CD4+T cells was measured; B: IL-2 secreted by CD4+T cells was measured (n=5). Experiments were performed at least three times independently. One-way ANOVA was used for Tukey's multiple comparison test. Experimental data are expressed as Mean ± SEM.

Supplementary Figure 5 Detection of the RBD-specific IgG1 and IgG2a titers elicited by TLR agonist-adjuvanted SARS-CoV-2 nanoparticle vaccine

(A) RBD-specific IgG1 titer in sera were measured by ELISA at week 6 (n=5). (B) RBD-specific IgG2a titer in sera were measured by ELISA at week 6 (n=5). Experiments were performed at least three times independently. One-way ANOVA was used for Tukey's multiple comparison test. Experimental data are expressed as Mean ± SEM.

Supplementary Figure 6 Transcriptional characterization of Pam2CSK4-adjuvanted SARS-CoV-2 nanoparticle vaccine

(A and B) Volcano plot of differential genes from peripheral leukocytes in the RBD-NP and RBD-NP+Pam2CSK4 groups of mice, respectively. A: three days after immunization; B: 7 days after immunization.

Supplementary Material

RNA-sequencing datasets

All RNA-seq data have been deposited in the Sequence Read Archive (SRA) under accession number PRJNA857815. Additional information and materials will be made available upon request.

Supplementary Figure 1

A

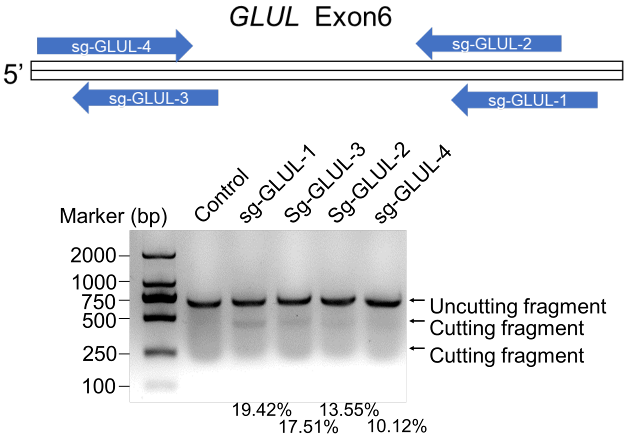

B

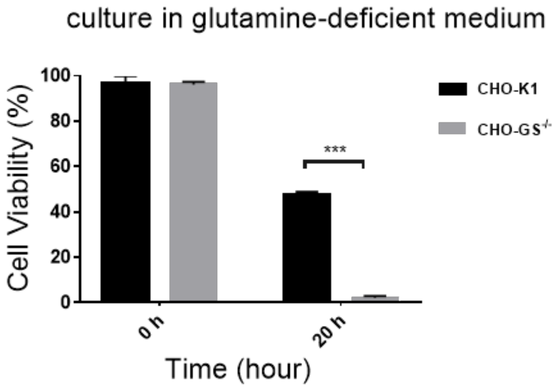

Supplementary Figure 2

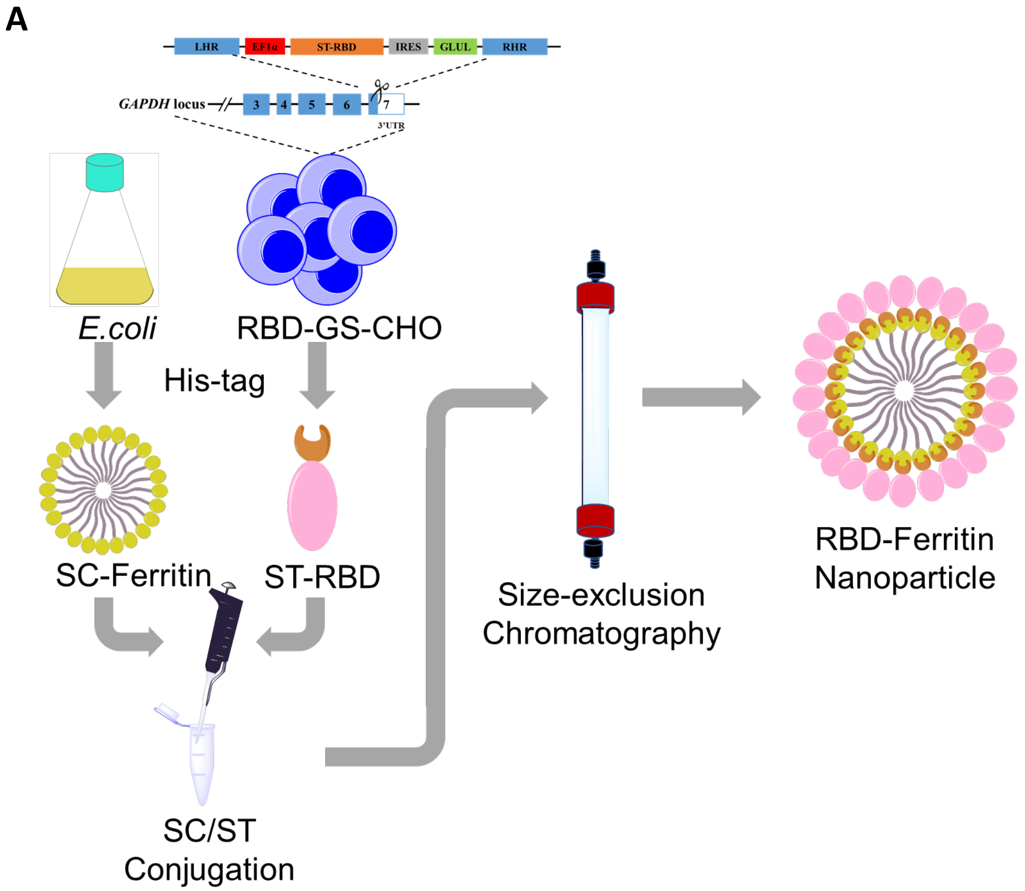

A

RBD-specific IgG LLPC of week 10

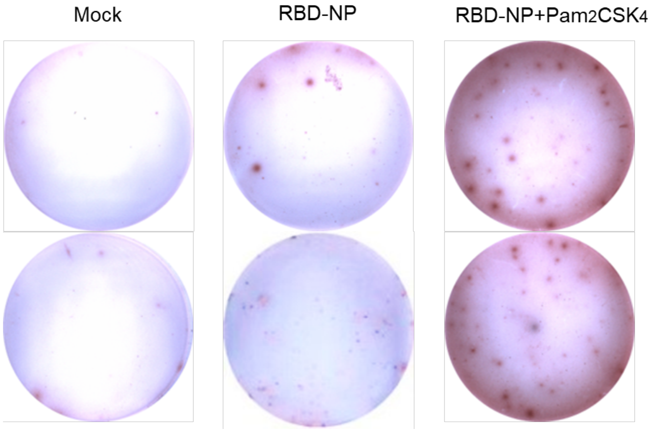

Supplementary Figure 4

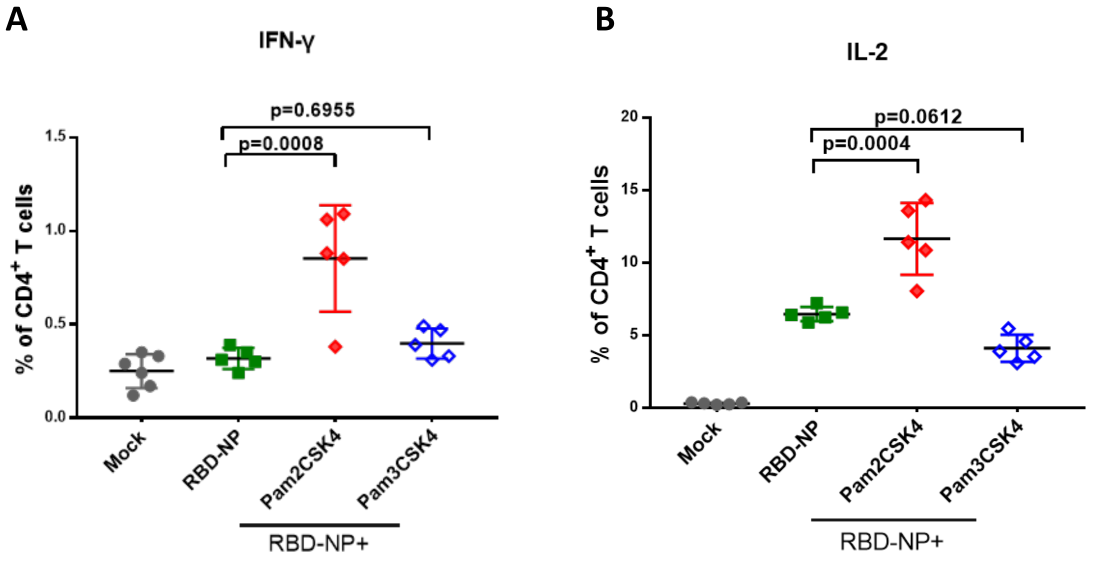

Supplementary Figure 5

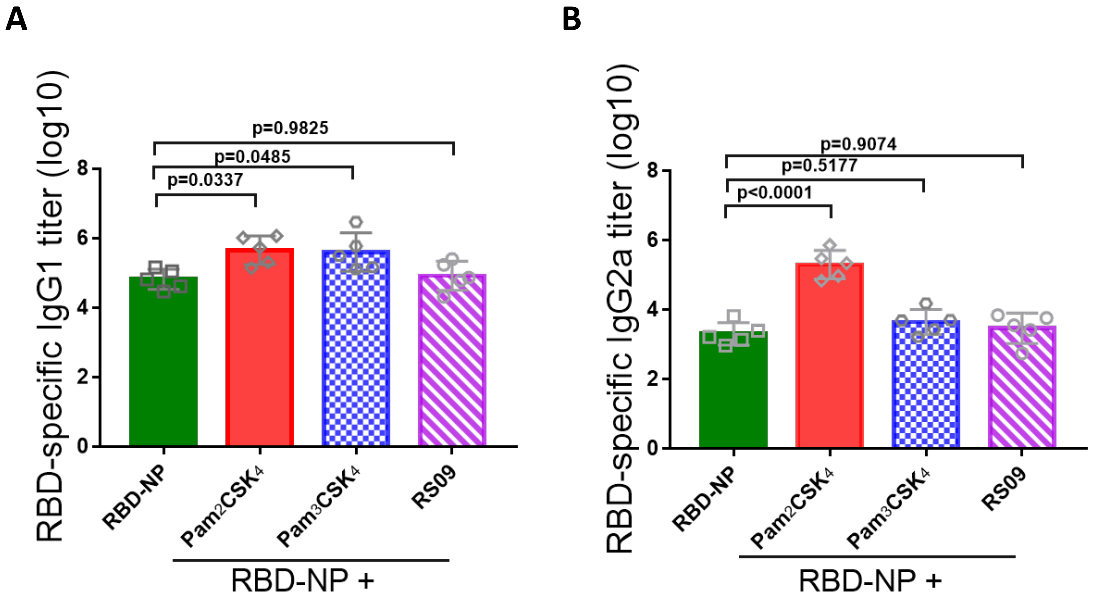

Supplementary Figure 6

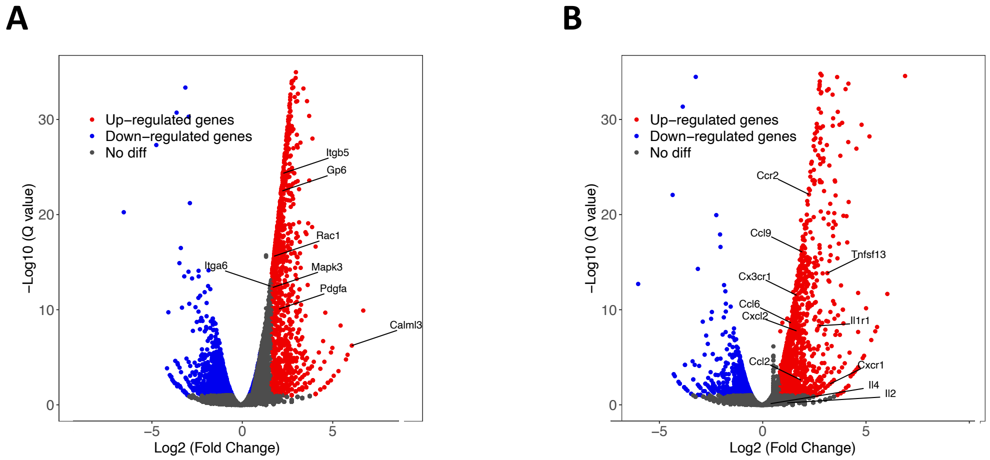

Supplement: Supplementary file 1 [file DataSheet_1.pdf]
